# New energy vehicle battery recycling strategy considering carbon emotion from a closed-loop supply chain perspective

**DOI:** 10.1038/s41598-024-51294-2

**Published:** 2024-01-06

**Authors:** Rong Guo, Yongjun He, Xianjun Tian, Yixin Li

**Affiliations:** 1 School of China Alcoholic Drinks, Luzhou Vocational and Technical College, Luzhou, 646000 China; 2 Intelligent Policing and National Security Risk Management Laboratory, Sichuan Police College, Luzhou, 646000 China; 3 School of Management, Xi’an University of Science and Technology, Xi’an, 710054 China

**Keywords:** Evolutionary theory, Environmental sciences, Engineering, Mathematics and computing

## Abstract

The negative impact of used batteries of new energy vehicles on the environment has attracted global attention, and how to effectively deal with used batteries of new energy vehicles has become a hot issue. This paper combines the rank-dependent expected utility with the evolutionary game theory, constructs an evolutionary game model based on the interaction mechanism between decision makers' emotions and decision making, and studies the recycling strategy of new energy automobile trams under the heterogeneous combination of emotions. The study shows that: (1) In addition to the establishment of effective external norms, the subjective preference of decision makers can also positively affect the recycling strategy of new energy vehicle batteries. (2) Fairness preferences can have a significant nonlinear effect on new energy vehicle battery recycling strategies by changing the utility function of decision makers. (3) When new energy vehicle manufacturers remain optimistic and new energy vehicle demanders remain rational or pessimistic, the new energy vehicle battery recycling strategy can reach the optimal steady state.

## Introduction

The transition from traditional energy to clean energy is the way to cope with the severe carbon emission reduction situation and achieve sustainable development. As a representative clean choice, new energy vehicles are gradually replacing the use of fuel vehicles due to the advantages of less pollution and high energy efficiency^1–3^. Driven by environmental requirements and encouraging policies, the new energy vehicle industry has made great progress in the past decade, especially in China^4,5^.

According to statistics released by the International Energy Agency, the global inventory of new energy vehicles has grown significantly from 14.97 million units in 2010 to 7.16783 million units in 2019, with a compound annual growth rate of 116.28%. In China, the cumulative inventory of new energy vehicles is 2,306,300 units, accounting for 45% of the global inventory. In addition, the production and sales volume of new energy vehicles increased significantly from 17.53 to 17.64 million units in 2013 to 12.42 and 12.06 million units in 2019, with a CAGR of 103.41 and 102.21%, respectively. At the same time, the penetration rate of new energy vehicle production and sales (the ratio of new energy vehicle production and sales to total vehicles) increased from 0.08% and 0.10% in 2013 to 4.83% and 4.68% in 2019, with compound annual growth rates of 98.07 and 89.83%, respectively. Power battery is the core component of new energy electric vehicles, and its average life is about 8 years^6,7^, which means that new energy electric vehicles, which have been produced on a large scale since 2014 and have exploded in growth, will usher in the "end-of-life wave" of power batteries in recent years^8^, and the domestic power battery retirement volume is expected to reach 708,000 in 2030^9^. In this context, power battery recycling recovery has become an important part of the sustainable development of the new energy vehicle industry^10^. The recycling of used power batteries is not only related to the response to the waste crisis, sustainable use of resources and environmental protection^11,12^, but also the key to effectively alleviate the challenges of scarce resources such as nickel, lithium, cobalt and manganese under the trend of cobalt-rich nickel^13,14^.

New energy battery recycling is a complex system engineering involving multiple participating subjects and multiple key links. Evolutionary game theory provides a systematic and effective research framework for studying new energy battery recycling due to its ability to portray the dynamic process of adaptive adjustment of decision makers' strategies over time^15^.Wei et al.^16^ constructed a three-party evolutionary game model under finite rationality conditions to analyze the governance strategies of end-of-life electric vehicle battery recycling, and found that in the early stage of battery recycling, penalty Zhang et al.^17^ modeled three parties including government, manufacturing and consumers based on an evolutionary game model in order to solve the difficulties in the reuse process of used batteries, and studied the problem of used battery resourcing under the subsidy reduction scenario. he et al.^18^ explored the supply-side perspective based on game theory, and explored the EPR mechanism of power battery recycling, and the study found that dynamic reward and punishment mechanism can make consumers and nearly 97% of new energy vehicle manufacturers participate in the environmental protection of power battery recycling. Guo et al.^19^ conducted a game analysis of power battery recycling in China based on the context of the contradiction between supply and demand of key metals, and found that the cost of cooperation will be a key factor affecting power battery recycling. In general, academic research results on new energy battery recycling are fruitful, but there are still studies that are worth further exploration. Many scholars have shown that decision makers have different emotional attitudes due to the differences in values and interests of decision makers^20^, and the information processing, decision making and behavior of decision makers are largely influenced and guided by emotions and emotions^21^, and the emotions of decision makers will then have a significant impact on the system operation. Traditional game analysis methods cannot effectively explain the influence of heterogeneous emotions of decision makers on behavior evolution due to their own limitations, which in turn makes the scientific and rigorous interpretation of game results urgent to be improved.

In terms of the influence of emotions on low-carbon behavior, human emotional responses to global carbon emissions, carbon emission reductions, and information in the field of climate change can potentially influence their low-carbon behavior^22^. Domestic and international scholars have begun to explore how micro-subjects' emotional responses to carbon emissions and climate change (referred to as carbon emotions in this paper) affect their carbon reduction behaviors from the perspective of behavioral economics. The results show that carbon sentiment determines the low-carbon behavior of government, enterprises and citizens by influencing people's psychological distance to carbon emissions^23^. As finite rational individuals^24^, the strategy choice of each participant in the new energy battery recycling process is not always theoretically optimal, and the new energy battery recycling strategy is also influenced by the carbon sentiment of manufacturers, retailers, and other participants. Therefore, it is necessary to consider the carbon sentiment of each participant in the new energy battery recycling process into the new energy battery recycling game model.

Rank Dependent Expected Utility (RDEU) theory takes into account the influence of participants' emotions on decision-making behavior and incorporates subjective emotional indicators into the modeling process, which makes up for the deficiency of traditional game theory that does not take enough account of realistic emotions^25^. Regarding this theoretical approach, existing studies have focused on wastewater treatment^26^, energy structure transformation^27^, manufacturing resource sharing^28^, and trade conflicts^29^, but there is limited research on how emotional factors affect new energy battery recycling.

The above discussion shows that the recycling of used batteries for new energy vehicles is significant, and it is crucial to discuss the recycling decisions and synergistic decisions of new energy vehicle manufacturers and retailers. In this paper, we are interested in how new energy vehicle manufacturers and retailers should make decisions to maximize their own benefits while taking into account the environmental benefits. In particular, this paper focuses on the following research questions:

1. Under what circumstances is recycling batteries an optimal strategy for new energy vehicle manufacturers?
2. Under what circumstances will new energy vehicle retailers choose to participate in the collaborative recycling of used batteries?
3. Under limited rationality, under what circumstances is the effect of carbon sentiment on new energy battery recycling strategies facilitative? Under what circumstances is it inhibiting?
4. How can policy designers design incentives to influence the battery recycling strategies of new energy vehicle manufacturers and retailers based on designing incentives?

In order to answer these questions, this paper constructs a two-party game model based on a closed-loop supply chain perspective, analyzes the behavioral decisions of manufacturers and retailers in the process of new energy battery recycling, explores the key parameters affecting new energy battery recycling, and then provides practical guidance for new energy battery recycling.

## Game model construction

### Theoretical basis

#### Evolutionary game theory

Classical game theory has been questioned by academics about the credibility of its results due to its own limitations such as the difficulty of solving Nash equilibrium, the irrationality of the assumption of complete rationality of decision makers, and the inability to solve dynamic multiple game equilibrium^30^. By drawing on biological evolution and its behavioral laws, Maynard proposed the idea of evolutionary games on the basis of classical game theory in 1973^31^, and in the subsequent research, in order to enhance the dynamics of evolutionary game theory so as to make it more relevant to the actual situation, Taylor proposed the replicator dynamics in 1978^32^. Nowadays, due to its applicability in portraying the pattern of change in decision makers' behavior in multi-period and long-stage processes, evolutionary game theory has been widely used in many fields such as supply chain management^33^, security management^34^, energy conservation and emission reduction^35^, PPP project^36^, and cooperative innovation^37^, etc., and a lot of research results have been achieved.

#### Rank-dependent expected utility theory

Rank-dependent expected utility (RDEU) theory, first proposed by Quiggin^38^, is a utility theory that takes into account the psychological preferences and emotions of decision makers. Under the conditions of decision uncertainty as well as high randomness, a real-valued function C defined by a utility function $U(x)$ and a decision weight function $\pi (x_i)$ is used to represent the degree of decision makers' preferences for different decisions, i.e., $V(x,u,\pi ) = \sum\limits_{i = 1}^{n} {\pi (x_i} )U(x_i),i = 1,2,3, \ldots ,n$.

For the set of strategies $X = \left\{ {x_i;i = 1,2, \ldots n} \right\}$, $P = \left\{ {X = xi} \right\} = p_i$. Assuming that strategy $x_i$ is ranked according to the size of utility function $U(x)$ and specifying $x_1 > x_2 > \cdots > x_n$, the utility rank of strategy $x_i$ is defined as $RP_i$. The probability distribution function of the strategy is therefore $RP_i = P(X \le x_i) = \sum\nolimits_{\tau \ge i}^{n} {p_i} ,i = 1,2, \ldots n$. Therefore, the larger the utility of the strategy, the larger its cumulative probability, and accordingly the greater the weight of the utility of the strategy in the decision.

The expression of the decision weight function is $\pi (x){ = }\omega (p_i + 1 - RP_i) - \omega (1 - RP_i)$. Where $\omega (\square)$ is a sentiment function and $\omega (\square)$ is a monotonically increasing function that satisfies $\omega (0){ = }0,\omega (1){ = }1$.

The function $\omega (\square)$ can be used to amplify or reduce the probability of $X \le x$.

There are three scenarios as follows:

1. when $\omega (p) < p$, $\omega (\square)$ is a concave function and $\omega (\square)$ will reduce the likelihood of $X \le x$, indicating the pessimism of the participant;
2. When $\omega (p) > p$, $\omega (\square)$ is a convex function and $\omega (\square)$ will magnify the likelihood of $X \le x$, indicating the optimism of the participant;
3. When $\omega (p){ = }p$, the likelihood of $X \le x$ is neither enlarged nor reduced, indicating the rationality of the participants.

RDEU theory treats decision weights in a nonlinear way, extending the expected utility theory in traditional game theory. This approach can better portray the influence of uncertainty in the decision-making environment and the limited rationality characteristics of the decision maker on the behavioral evolution, and to a certain extent, make up for the research gap of traditional game theory in the emotional dimension.

### Model construction

The specific parameters of the evolutionary game model of new energy battery recycling are set as shown in Table 1:

**Table 1:** Parameter symbols and their meanings.

Parameter	Meaning
$x$	Probability of aggressive battery recycling strategies by new energy vehicle manufacturers
$y$	Possibility of new energy vehicle retailers choosing Active synergy
$C_{x}$	Cost for new energy vehicle manufacturers to adopt an aggressive battery recycling strategy
$C_{y}$	Cost of active synergy for new energy vehicle retailers
$L_{a}$	Base benefit for new energy vehicle manufacturers
$L_b$	Base benefit for new energy vehicle retailers
$K_{a}$	Altruistic preferences of new energy vehicle manufacturers
$K_{b}$	Altruistic preferences of new energy vehicle retailers
$H_{a}$	Marginal gain from battery recycling
$H_{b}$	"Pass-through" benefits due to spillover effects
$D$	Peer incentives
$F$	Peer penalties
$M$	Positive consumer feedback
$N$	Negative consumer feedback
$L$	Quality risk
$\psi$	Risk Factor
$\varphi$	Risk transmission coefficient
$q$	Probability of risk occurrence
$r_{1}$	Emotional intensity of new energy vehicle manufacturers
$r_{2}$	Emotional intensity of new energy vehicle retailers

### key assumptions

In order to better construct the three-party evolutionary game model, the key assumptions of this paper are shown below:

#### Key assumption 1

Consider a secondary new energy vehicle closed-loop supply chain consisting of a single new energy vehicle manufacturer and a single new energy vehicle retailer. The new energy vehicle manufacturer produces new energy vehicles and processes the recycled used batteries to obtain remanufactured batteries, after which the remanufactured batteries are used to produce new energy vehicles and wholesale the entire vehicle to the new energy vehicle retailer, which eventually sells it to consumers. The strategy choice of new energy vehicle manufacturers can be divided into (positive battery recycling, negative battery recycling). The strategy choice of new energy vehicle retailers can be divided into (positive synergy, negative synergy). Each subject continuously adjusts its own strategy in the game process, until the strategy evolution reaches the equilibrium state.

#### Key assumption 2

In the process of new energy vehicle battery recycling, each participant will show irrational state and carbon sentiment will influence the battery recycling decisions of new energy vehicle manufacturers and new energy vehicle retailers. The carbon sentiment intensity of new energy vehicle manufacturers and new energy vehicle retailers is represented by $r_{1}$ and $r_{2}$, respectively. According to the RDEU theory, under the influence of emotion, the subjective probability function becomes $\omega (p){ = }p^{{\text{r}}}$, and $p$($0 \le p \le 1$) represents the objective probability of decision occurrence. When $r_{i} = 1$, the subjective probability value is the same as the objective probability value, the game subject does not have emotion and is in rational state; when $r_{i} < 1$, the subjective probability value is higher than the objective probability value, the game subject overestimates the choice probability and shows optimism; when $r_{i} > 1$, the subjective probability value is lower than the objective probability value, the game subject underestimates the choice probability and shows pessimism.

#### Key assumption 3

The production and sale of the product will generate the base revenue, with $L_{a}$ and $L_b$ denoting the base revenue of the new energy vehicle manufacturer and the new energy vehicle retailer, respectively. The positive battery recycling cost of the new energy vehicle manufacturer is $C_{x}$ and the positive synergy cost of the new energy vehicle retailer is $C_{y}$. $K_{a}$ and $K_{b}$ denote the altruistic preferences of the new energy vehicle manufacturer and the new energy vehicle retailer, respectively. d denotes the marginal benefit of battery recycling, and for the convenience of calculation, it is assumed that the new energy vehicle manufacturer and the new energy vehicle retailer do not incur costs when they adopt negative strategies. In the process of new energy battery recycling, there is the phenomenon of “free-rider” due to the spillover effect, and the “free-rider” benefit due to the spillover effect is denoted by $H_{b}$. In order to better motivate new energy vehicle manufacturers and new energy vehicle retailers to actively participate in battery recycling, all node enterprises in the closed-loop supply chain will contribute to a peer incentive fund pool to motivate new energy vehicle manufacturers and new energy vehicle retailers with positive battery recycling, and the incentive amount will be $D$. At the same time, new energy vehicle manufacturers with negative battery recycling and new energy vehicle retailers with negative collaboration will be punished. The penalty amount is $F$. $D$ and $F$ are fixed values.

#### Key assumption 4

Consumers, as end users of new energy vehicle products, are also affected by the decisions of new energy vehicle manufacturers and new energy vehicle retailers. Retired power batteries generally have 70–80% of their initial capacity and still have great economic value. It is assumed that when new energy vehicle manufacturers actively recycle batteries and new energy vehicle retailers actively cooperate, the quality of the remanufactured vehicles produced will be the same as that of new energy vehicles, and consumers will have a better consumption experience, which in turn will generate positive feedback to new energy vehicle manufacturers and new energy vehicle retailers, and the positive feedback utility of consumers is represented by $M$. Conversely, quality risk will occur with a probability of $q$, denoted by $\psi$ as the risk factor. According to the risk sharing principle, when one party chooses positive behavior and the other party chooses negative behavior, the quality risk $L$ will be transferred from one party to the other party according to the risk transfer coefficient $\varphi$. In this case, consumers will have the negative feedback to the new energy vehicle manufacturers and new energy vehicle retailers because of the negative "spillover" and get a poorer consumption experience. The negative feedback utility of consumers is represented by $N$.

### Revenue matrix

Based on the above discussion, the evolutionary game payment matrix of new energy vehicle battery recycling is shown in Table 2.

**Table 2:** Revenue Matrix.

Participating parties	New energy vehicle retailers	
	Positive synergy $y$	Negative synergy $1 - y$
New energy vehicle manufacturers		
Positive battery recycling	$L_{a} - C_{x} + K_{a}H_{a} + D + M + K_{b}H_{b}$	$L_{a} - C_{x} + K_{a}H_{a} + D + M - \psi Lq$
$x$	$L_b - C_{y} + K_{b}H_{a} + D + M + K_{a}H_{b}$	$L_b - F - N + K_{a}H_{b} - \phi \psi Lq$
Negative battery recycling	$L_{a} + K_{b}H_{b} - F - N - \phi \psi Lq$	$L_{a} - F - N - Lq$
$1 - x$	$L_b - C_{y} + K_{b}H_{a} + D + M - \psi Lq$	$L_b - F - N - Lq$

## Game analysis

Based on the above model assumptions and RDEU theory, the hierarchy-dependent expected utility models of new energy vehicle manufacturers and new energy vehicle retailers with different strategies are constructed.

### Strategy stability analysis of new energy vehicle manufacturers

For the new energy vehicle manufacturer, the utility ranking corresponding to its four strategy choices, based on the relationship between the parameters of costs, benefits, incentives, and penalties, is:

$$\begin{aligned} & L_{a} - C_{x} + K_{a}H_{a} + D + M + K_{b}H_{b} > L_{a} - C_{x} + K_{a}H_{a} + D + M - \psi Lq \\ & > L_{a} + K_{b}H_{b} - F - N - \varphi \psi Lq > L_{a} - F - N - Lq \\ \end{aligned}$$

The utility, probability, rank and decision weights corresponding to each strategy of the new energy vehicle manufacturer are shown in Table 3.

**Table 3:** Expected utility of new energy vehicle manufacturer rank dependence considering emotions.

New energy vehicle manufacturers effectiveness	Probability	Rank	Decision weight
$L_{a} - C_{x} + K_{a}H_{a} + D + M + K_{b}H_{b}$	$xy$	$1$	$\omega_{A}(xy)$
$L_{a} - C_{x} + K_{a}H_{a} + D + M - \psi Lq$	$x(1 - y)$	$1 - xy$	$\omega_{A}(x) - \omega_{A}(xy)$
$L_{a} + K_{b}H_{b} - F - N - \varphi \psi Lq$	$(1 - x)y$	$1 - x$	$\omega_{A}(x + y - xy) - \omega_{A}(x)$
$L_{a} - F - N - Lq$	$(1 - x)(1 - y)$	$1 - x - y + xy$	$1 - \omega_{A}(x + y - xy)$

The expected benefit of “positive battery recycling” for new energy vehicle manufacturers is:

1 $$\begin{aligned} & U_{1x} = [L_{a} - C_{x} + K_{a}H_{a} + D + M + K_{b}H_{b}]y^{r_{2}} + [L_{a} - C_{x} + K_{a}H_{a} + D + M - \psi Lq](1 - y^{r_{2}} ) \\ & = L_{a} - C_{x} + K_{a}H_{a} + D + M - (1 - y^{r_{2}} )\psi Lq{ + }K_{b}H_{b}y^{r_{2}} \\ \end{aligned}$$

The expected benefit of choosing “negative battery recycling” for new energy vehicle manufacturers is:

2 $$\begin{aligned} & U_{2x} = (L_{a} + K_{b}H_{b} - F - N - \varphi \psi Lq)y^{r_{2}} + (L_{a} - F - N - Lq)(1 - y^{r_{2}} ) \\ & = L_{a} - F - N + K_{b}H_{b}y^{r_{2}} - (1 + \varphi \psi y^{r_{2}} - y^{r_{2}} )Lq \\ \end{aligned}$$

The average expected benefit of the strategy choice for new energy vehicle manufacturers is:

3 $$\begin{aligned} &\overline{U_{x}} = (L_{a} - C_{x} + K_{a}H_{a} + D + M + K_{b}H_{b})\omega_{A}(xy) + (L_{a} - C_{x} + K_{a}H_{a} + D + M - \psi Lq)[\omega_{A}(x) - \omega_{A}(xy)] \hfill \\& + (L_{a} + K_{b}H_{b} - F - N - \varphi \psi Lq)[\omega_{A}(x + y - xy) - \omega_{A}(x)] + (L_{a} - F - N - Lq)[1 - \omega_{A}(x + y - xy)] \hfill \\ &= (K_{b}H_{b}{ + }\psi Lq)(xy)^{r_{1}} + ( - C_{x} + K_{a}H_{a} + D + M - \psi Lq - K_{b}H_{b} + F + N + \varphi \psi Lq)x^{r_{1}} \hfill \\ & + (K_{b}H_{b} - \varphi \psi Lq + Lq)(x + y - xy)^{r_{1}} + L_{a} - F - N - Lq \hfill \\ \end{aligned}$$

The replicated dynamic equation for the new energy vehicle manufacturer is

4 $$\begin{aligned} & F(x) = {{dx} \mathord{\left/ {\vphantom {{dx} {dt = }}} \right. \kern-0pt} {dt = }}x^{{{\text{r}}1}} (U_{1x} - \overline{U_{x}} ) = x^{{{\text{r}}1}} [F + N + Lq - C_{x} + K_{a}H_{a} + D + M \\ &\quad - (1 - y^{r_{2}} )\psi Lq{ + }K_{b}H_{b}y^{r_{2}} - (K_{b}H_{b}{ + }\psi Lq)(xy)^{r_{1}} - ( - C_{x} + K_{a}H_{a} + D \\ &\quad+ M - \psi Lq - K_{b}H_{b} + F + N + \varphi \psi Lq)x^{r_{1}} - (K_{b}H_{b} - \varphi \psi Lq + Lq)(x + y - xy)^{r_{1}} ] \\ \end{aligned}$$

From Eq. (4), it can be obtained that when $x = 0$, $x = 1$ or $x = x^{*}$, the new energy vehicle manufacturer can achieve local stability by choosing an aggressive battery recycling strategy.

### New energy vehicle retailer strategy stability analysis

For the new energy vehicle retailers, the utility ranking corresponding to its four strategy choices, based on the relationship between the parameters of costs, benefits, incentives, and penalties, is:

$$\begin{aligned} & L_b - C_{y} + K_{b}H_{a} + D + M + K_{a}H_{b} > L_b - C_{y} + K_{b}H_{a} + D + M - \psi Lq \\ & > L_b - F - N + K_{a}H_{b} - \varphi \psi Lq > L_b - F - N - Lq \\ \end{aligned}$$

The utility, probability, rank and decision weights corresponding to each strategy of the new energy vehicle retailers are shown in Table 4.

**Table 4:** Expected utility of new energy vehicle retailer rank dependence considering emotions.

New Energy Vehicle Retailers Utility	Probability	Rank	Decision weight
$L_b - C_{y} + K_{b}H_{a} + D + M + K_{a}H_{b}$	$xy$	$1$	$\omega_{B}(xy)$
$L_b - C_{y} + K_{b}H_{a} + D + M - \psi Lq$	$(1 - x)y$	$1 - xy$	$\omega_{B}(y) - \omega_{B}(xy)$
$L_b - F - N + K_{a}H_{b} - \varphi \psi Lq$	$x(1 - y)$	$1 - y$	$\omega_{B}(x + y - xy) - \omega_{B}(y)$
$L_b - F - N - Lq$	$(1 - x)(1 - y)$	$1 - x - y + xy$	$1 - \omega_{B}(x + y - xy)$

The expected benefits of choosing "positive synergy" for new energy vehicle retailers is:

5 $$\begin{aligned} U_{1y} &= [L_b - C_{y} + K_{b}H_{a} + D + M + K_{a}H_{b}]x^{r_{1}} + [L_b - C_{y} + K_{b}H_{a} + D + M - \psi Lq](1 - x^{r_{1}} ) \\ &\quad = L_b - C_{y} + K_{b}H_{a} + D + M - (1 - x^{r_{1}} )\psi Lq + K_{a}H_{b}x^{r_{1}} \\ \end{aligned}$$

The expected benefits of choosing "negative synergy" for new energy vehicle retailers is:

6 $$\begin{aligned} U_{2y} &= (L_b - F - N + K_{a}H_{b} - \varphi \psi Lq)x^{r_{1}} + (L_b - F - N - Lq)(1 - x^{r_{1}} ) \\ & \quad= L_b - F - N + K_{a}H_{b} - (1 + \varphi \psi x^{r_{1}} - x^{r_{1}} )Lq \\ \end{aligned}$$

The average expected benefit of the strategy choice of the new energy vehicle manufacturer is:

7 $$\begin{aligned} \overline{U_{y}} & = (L_b - C_{y} + K_{b}H_{a} + D + M + K_{a}H_{b})\omega_{B}(xy) + (L_b - C_{y} + K_{b}H_{a} + D + M - \psi Lq)[\omega_{B}(y) - \omega_{B}(xy)] \\ & \quad+ (L_b - F - N + K_{a}H_{b} + \varphi \psi Lq)[\omega_{B}(x + y - xy) - \omega_{B}(y)] + (L_b - F - N - Lq)[1 - \omega_{B}(x + y - xy)] \\ & = (K_{a}H_{b} + \psi Lq)(xy)^{r_{2}} + ( - C_{y} + K_{b}H_{a} + D + M - \psi Lq + F + N - K_{a}H_{b} + \varphi \psi Lq)(y)^{r_{2}} \\ & \quad+ (K_{a}H_{b} - \varphi \psi Lq + Lq)(x + y - xy)^{r_{2}} + L_b - F - N - Lq \\ \end{aligned}$$

The replicated dynamic equation for the new energy retailer is

8 $$\begin{aligned} & F(y) = {{dy} \mathord{\left/ {\vphantom {{dy} {dt = }}} \right. \kern-0pt} {dt = }}y^{{{\text{r}}2}} (U_{1y} - \overline{U_{y}} ) = y^{{{\text{r}}2}} [F + N + Lq - C_{y} + K_{b}H_{a} + D + M \\ & - (1 - x^{r_{1}} )\psi Lq + K_{a}H_{b}x^{r_{1}} - (K_{a}H_{b} + \psi Lq)(xy)^{r_{2}} - ( - C_{y} + K_{b}H_{a} + D + M \\ & - \psi Lq + F + N - K_{a}H_{b} + \varphi \psi Lq)(y)^{r_{2}} - (K_{a}H_{b} - \varphi \psi Lq + Lq)(x + y - xy)^{r_{2}} ] \\ \end{aligned}$$

From Eq. (8), it can be obtained that when $y = 0$, $y = 1$ or $y = y^{*}$, new energy vehicle retailers can achieve local stability by choosing an aggressive synergy strategy.

### Strategy combination stability analysis

From this “Strategy stability analysis of new energy vehicle manufacturers” and “New energy vehicle retailer strategy stability analysis” analysis, it can be seen that the five local equilibrium points of the evolutionary game model are $E_{1}(0,0)$, $E_{2}(0,1)$, $E_{3}(1,0)$, $E_{4}(1,1)$, $E_{5}(x^{*} ,y^{*} )$.

According to the stability analysis of the evolutionary game, the stability of the strategy combination of each game subject can be judged according to the Lyapunov indirect method, and the Jacobian matrix of the game model can be obtained from the formula (4) and (8) as follows:

9 $$J = \left[ \begin{gathered} \partial F(x)/\partial x \partial F(x)/\partial y \hfill \\ \partial F(y)/\partial x \partial F(y)/\partial y \hfill \\ \end{gathered} \right]$$

Among them:

10 $$\begin{aligned} \partial F(x)/\partial x & = r_{1}x^{{{\text{r}}1 - 1}} [F + N + Lq - C_{x} + K_{a}H_{a} + D + M - (1 - y^{r_{2}} )\psi Lq{ + }K_{b}H_{b}y^{r_{2}} - (K_{b}H_{b}{ + }\psi Lq)(xy)^{r_{1}} \\ &\quad - ( - C_{x} + K_{a}H_{a} + D + M - \psi Lq - K_{b}H_{b} + F + N + \varphi \psi Lq)x^{r_{1}} - (K_{b}H_{b} - \varphi \psi Lq + Lq)(x + y - xy)^{r_{1}} ] \\ &\quad + x^{{{\text{r}}1}} [ - (K_{b}H_{b}{ + }\psi Lq)r_{1}x^{{{\text{r}}1 - 1}} y^{r_{1}} - ( - C_{x} + K_{a}H_{a} + D + M - \psi Lq - K_{b}H_{b} + F + N + \varphi \psi Lq)r_{1}x^{{{\text{r}}1 - 1}} \\ &\quad -(K_{b}H_{b} - \varphi \psi Lq + Lq)r_{1}(1 - y)(x + y - xy)^{{{\text{r}}1 - 1}} ] \\ \end{aligned}$$

11 $$\begin{aligned} \partial F(y)/\partial y & = r_{2}y^{{{\text{r}}2 - 1}} [F + N + Lq - C_{y} + K_{b}H_{a} + D + M - (1 - x^{r_{1}} )\psi Lq + K_{a}H_{b}x^{r_{1}} - (K_{a}H_{b} + \psi Lq)(xy)^{r_{2}} \\ &\quad - ( - C_{y} + K_{b}H_{a} + D + M - \psi Lq + F + N - K_{a}H_{b} + \varphi \psi Lq)(y)^{r_{2}} - (K_{a}H_{b} - \varphi \psi Lq + Lq)(x + y - xy)^{r_{2}} ] \\ & \quad+ y^{{{\text{r}}2}} [ - (K_{a}H_{b} + \psi Lq)x^{r_{2}} r_{2}y^{r_{2} - 1} - ( - C_{y} + K_{b}H_{a} + D + M - \psi Lq + F + N - K_{a}H_{b} + \varphi \psi Lq)r_{2}y^{r_{2} - 1} \\ &\quad - (K_{a}H_{b} - \varphi \psi Lq + Lq)r_{2}(1 - x)(x + y - xy)^{r_{2} - 1} ] \\ \end{aligned}$$

12 $$\begin{aligned} & \partial F(x)/\partial y = x^{{{\text{r}}1}} [r_{2}y^{r_{2} - 1} \psi Lq{ + }r_{2}K_{b}H_{b}y^{r_{2} - 1} - r_{1}(K_{b}H_{b}{ + }\psi Lq)x^{r_{1}} y^{r_{1} - 1} \\ &\quad - r_{1}(K_{b}H_{b} - \varphi \psi Lq + Lq)(1 - x)(x + y - xy)^{r_{1} - 1} ] \\ \end{aligned}$$

13 $$\begin{aligned} & \partial F(y)/\partial x = y^{{{\text{r}}2}} [r_{1}x^{r_{1} - 1} \psi Lq + r_{1}K_{a}H_{b}x^{r_{1} - 1} - (K_{a}H_{b} + \psi Lq)r_{2}x^{r_{2} - 1} y^{r_{2}} \\ &\quad - (K_{a}H_{b} - \varphi \psi Lq + Lq)r_{2}(1 - y)(x + y - xy)^{r_{2} - 1} ] \\ \end{aligned}$$

14 $$Det(J) = (\partial F(x)/\partial x)(\partial F(y)/\partial y) - (\partial F(x)/\partial y)(\partial F(y)/\partial x)$$

15 $$Tr(J) = \partial F(x)/\partial x + \partial F(y)/\partial y$$

Since the values of Jacobian matrix are related to the values of model variables, the values of Jacobian matrix are different under different emotional states of game subjects, and thus the equilibrium points obtained are also different. Therefore, based on the different emotional states of game subjects, this paper analyzes the stability of strategy combinations of new energy vehicle manufacturers and new energy vehicle retailers in four contexts: (rational, rational), (emotional, emotional), (rational, emotional), and (emotional, rational), respectively.

#### Scenario 1: new energy vehicle manufacturers are rational, new energy vehicle retailers are rational

When the new energy vehicle manufacturer is rational and the new energy vehicle retailer is rational, the sentiment parameter $r_{1} = 1,r_{2} = 1$ at this time. The sentiment parameters are brought into each replication dynamic equation, at this time the strategy portfolio stability analysis is shown in Table 5.

**Table 5:** Stability analysis of strategy combinations under rational new energy vehicle manufacturer and rational new energy vehicle retailer scenarios.

Equilibrium point	$Det(J)$	$Tr(J)$	Stability
$E_{1}(0,0)$	$\times$	$\times$	Saddle point
$E_{2}(1,0)$	$\times$	$\times$	Saddle point
$E_{3}(0,1)$	$\times$	$\times$	Saddle point
$E_{4}(1,1)$	$\times$	$\times$	Saddle point
$E_{5}(x^{ * } ,y^{ * } )$	Stability depends on specific values		

As can be seen from Table 5, when the new energy vehicle manufacturer is rational and the new energy vehicle retailer is rational, there are four saddle points in the system, which are $E_{1}(0,0)$, $E_{2}(1,0)$, $E_{3}(0,1)$, and $E_{4}(1,1)$. The stability of the local equilibrium point $E_{5}(p^{*} ,e^{*} )$ also cannot be determined, and its stability depends on the specific value taken.

#### Scenario 2: new energy vehicle manufacturers are emotional, new energy vehicle retailers are emotional

When the new energy vehicle manufacturer sentiment, new energy vehicle retailer sentiment, at this time the sentiment parameter $r_{1} \ne 1,r_{2} \ne 1$. The sentiment parameters are brought into each replication dynamic equation, at this time the strategy portfolio stability analysis is shown in Table 6.

**Table 6:** Stability analysis of strategy combinations under emotional new energy vehicle manufacturer and emotional new energy vehicle retailer scenarios.

Equilibrium point	$Det(J)$	$Tr(J)$	Stability
$E_{1}(0,0)$	$0$	$0$	Unstable
$E_{2}(1,0)$	$0$	$\times$	Unstable
$E_{3}(0,1)$	$0$	$\times$	Unstable
$E_{4}(1,1)$	$\times$	$\times$	Saddle point
$E_{5}(x^{ * } ,y^{ * } )$	Stability depends on specific values and emotional intensity		

As can be seen from Table 6, when the new energy vehicle manufacturer emotion, the new energy vehicle retailer emotion, the system exists three instability points: $E_{1}(0,0)$, $E_{2}(1,0)$, $E_{3}(0,1)$, and a saddle point $E_{4}(1,1)$, at the same time, because the intensity of the new energy vehicle manufacturer and new energy vehicle retailer emotion is unknown, that is, the specific value of the emotion parameter $r_{1},r_{2}$ cannot be determined, so the stability of the local equilibrium point $E_{5}(p^{*} ,e^{*} )$ can not be determined, its stability depends on the specific value and emotion intensity.

#### Scenario 3: new energy vehicle manufacturers are rational, new energy vehicle retailers are emotional

When the new energy vehicle manufacturer is rational and the new energy vehicle retailer is emotional, at this time the emotional parameter $r_{1} = 1,r_{2} \ne 1$. Bringing the emotional parameter into each replication dynamic equation, at this time the strategy portfolio stability analysis is shown in Table 7.

**Table 7:** Stability analysis of strategy combinations under rational new energy vehicle manufacturer and emotional new energy vehicle retailer scenarios.

Equilibrium point	$Det(J)$	$Tr(J)$	Stability
$E_{1}(0,0)$	$0$	$\times$	Unstable
$E_{2}(1,0)$	$0$	$\times$	Unstable
$E_{3}(0,1)$	$\times$	$\times$	Saddle point
$E_{4}(1,1)$	$\times$	$\times$	Saddle point
$E_{5}(x^{ * } ,y^{ * } )$	Stability depends on specific values and emotional intensity		

As can be seen from Table 7, when the new energy vehicle manufacturer is rational and the new energy vehicle retailer is emotional, the system has two instability points $E_{1}(0,0)$, $E_{2}(1,0)$, and two saddle points $E_{3}(0,1)$, $E_{4}(1,1)$. At the same time, the stability of the local equilibrium point $r_{2}$ cannot be determined because the emotional intensity of the new energy vehicle retailer is unknown, i.e., the specific value of the emotional parameter $E_{5}(p^{*} ,e^{*} )$ cannot be determined, and its stability depends on the specific value and emotional intensity.

#### Scenario 4: new energy vehicle manufacturers are emotional, new energy vehicle retailers are rational

When the new energy vehicle manufacturer sentiment, new energy vehicle retailers rational, at this time the sentiment parameter $r_{1} \ne 1,r_{2} = 1$. The sentiment parameter is brought into each replication dynamic equation, at this time the strategy portfolio stability analysis is shown in Table 8.

**Table 8:** Stability analysis of strategy combinations under rational new energy vehicle manufacturer and rational new energy vehicle retailer scenarios.

Equilibrium point	$Det(J)$	$Tr(J)$	Stability
$E_{1}(0,0)$	$0$	$\times$	Unstable
$E_{2}(1,0)$	$\times$	$\times$	Saddle point
$E_{3}(0,1)$	$0$	$\times$	Unstable
$E_{4}(1,1)$	$\times$	$\times$	Saddle point
$E_{5}(x^{ * } ,y^{ * } )$	Stability depends on specific values and emotional intensity		

As can be seen from Table 8, when the new energy vehicle manufacturer is emotional and the new energy vehicle retailer is rational, the system has two instability points $E_{1}(0,0)$ and $E_{3}(0,1)$, and two saddle points $E_{2}(1,0)$, $E_{4}(1,1)$. At the same time, the stability of the local equilibrium point $r_{1}$ cannot be determined because the emotional intensity of the new energy vehicle manufacturer is unknown, i.e., the specific value of the emotional parameter $E_{5}(p^{*} ,e^{*} )$ cannot be determined, and its stability depends on the specific value and emotional intensity.’

## Simulation analysis

In order to more intuitively analyze the evolutionary law between the new energy vehicle manufacturer and the new energy vehicle retailer, the key parameters and the influence of heterogeneous emotions on the evolutionary stability are studied. In this paper, MATLAB is used for simulation analysis, and the parameters are set with reference to the literature^15,28,30^ and adjusted according to the opinions of experts in related fields, and the specific parameter settings are shown in Table 9.

**Table 9:** Parameter settings.

Parameter	$x$	$y$	$C_{x}$	$C_{y}$	$L_{a}$	$L_b$	$K_{a}$	$K_{b}$	$H_{a}$	$H_{b}$
Initial Value	0.4	0.4	4	4	5.5	5	0.4	0.3	2	2
Parameter	$D$	$F$	$M$	$N$	$L$	$\psi$	$\phi$	$q$	$r_{1}$	$r_{2}$
Initial Value	1	1	0.8	0.5	1	0.2	0.2	0.5	1	1

### Influence of key parameters

#### Influence of altruistic preferences

**Figure 1:**
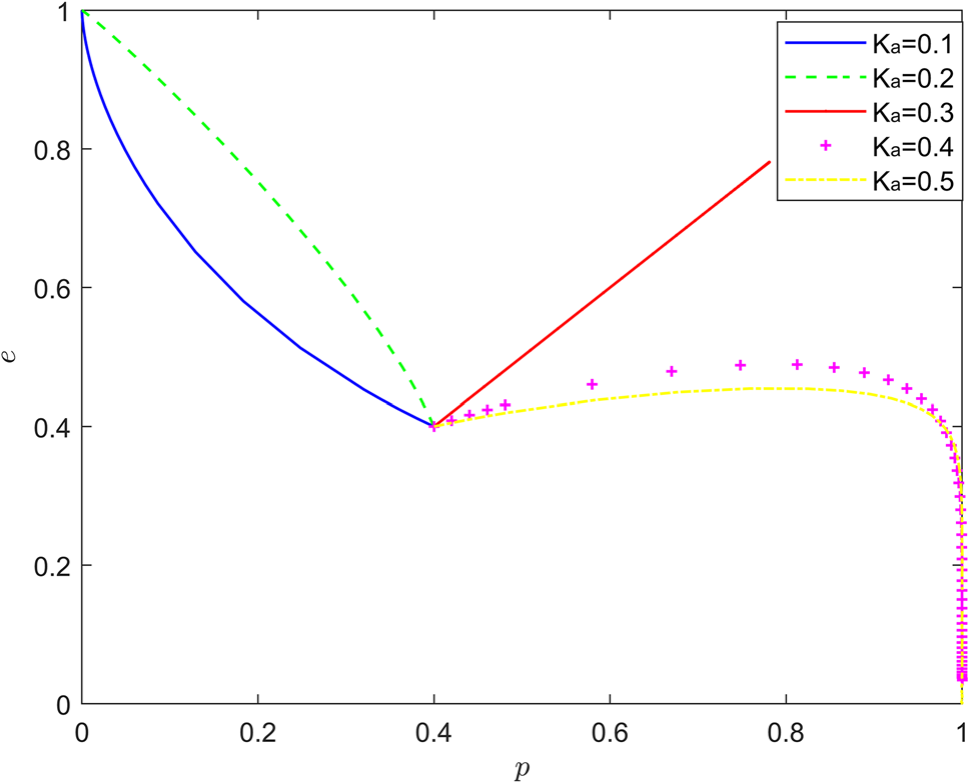
Effect of $K_{a}$ on the evolution of the system.

Taking $\left\{ {K_{a} = 0.1,K_{a} = 0.2,K_{a} = 0.3,K_{a} = 0.4,K_{a} = 0.5} \right\}$, the strategy evolution process and results are shown in Fig. 1.

**Figure 2:**
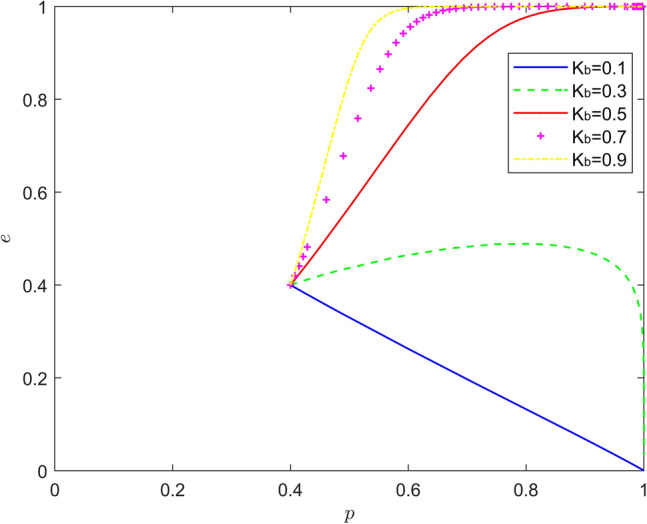
Effect of $K_{b}$ on the evolution of the system.

Taking $\left\{ {K_{b} = 0.1,K_{a} = 0.3,K_{a} = 0.5,K_{a} = 0.7,K_{a} = 0.9} \right\}$, the strategy evolution process and results are shown in Fig. 2.

As can be seen from Figs. 1 and 2, there is a nonlinear relationship between altruistic preference of new energy vehicle manufacturers and new energy vehicle retailers and battery recycling. When the altruistic preference of new energy automobile manufacturers is too low, the probability of battery recycling will remain at a low level, and when the altruistic preference of new energy automobile manufacturers is too high, the "hitchhiking" behavior of new energy automobile retailers will intensify, resulting in the deterioration of battery recycling effect of new energy automobile manufacturers with the enhancement of altruistic preference; When the altruistic preference of new energy vehicle retailers is too low, the probability of participating in battery recycling will remain at a low level. When the altruistic preference of new energy vehicle manufacturers is too high, battery recycling will be inflexible, and the battery recycling effect of new energy vehicles will not change with the change of altruistic preference. This nonlinear feature makes the altruistic preference of new energy vehicle manufacturers and new energy vehicle retailers only in a moderate range to better play its positive effect on battery recycling of new energy vehicles.

#### Influence of peer mechanism

**Figure 3:**
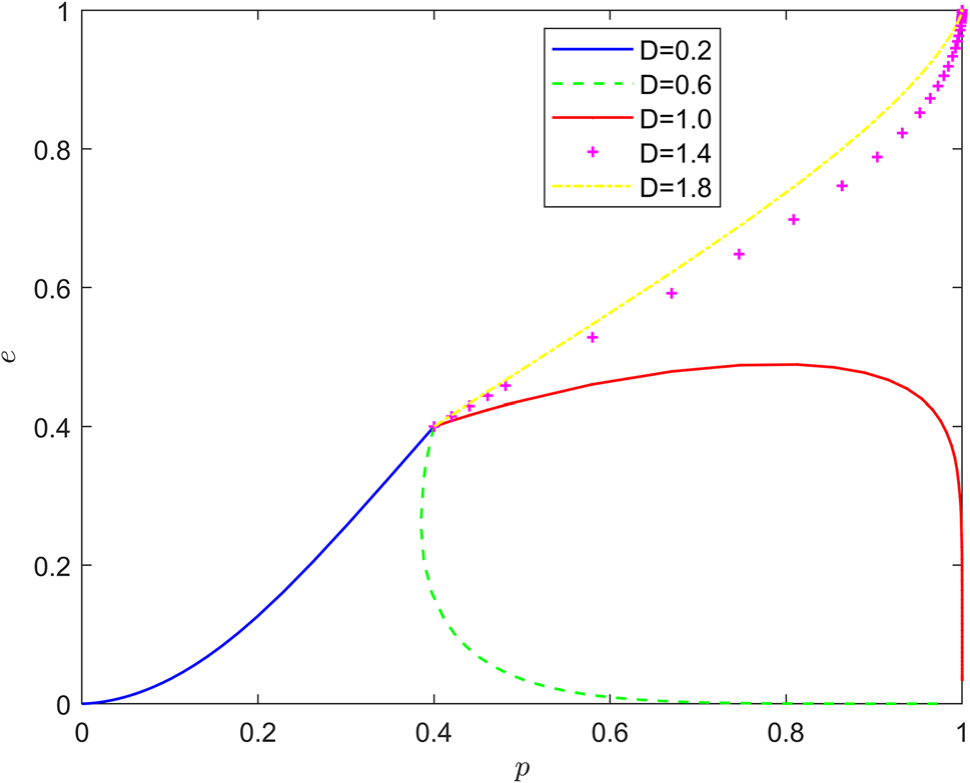
Effect of $D$ on the evolution of the system.

Taking $\left\{ {D = 0.2,D = 0.6,D = 1.0,D = 1.4,D = 1.8} \right\}$, the strategy evolution process and results are shown in Fig. 3.

**Figure 4:**
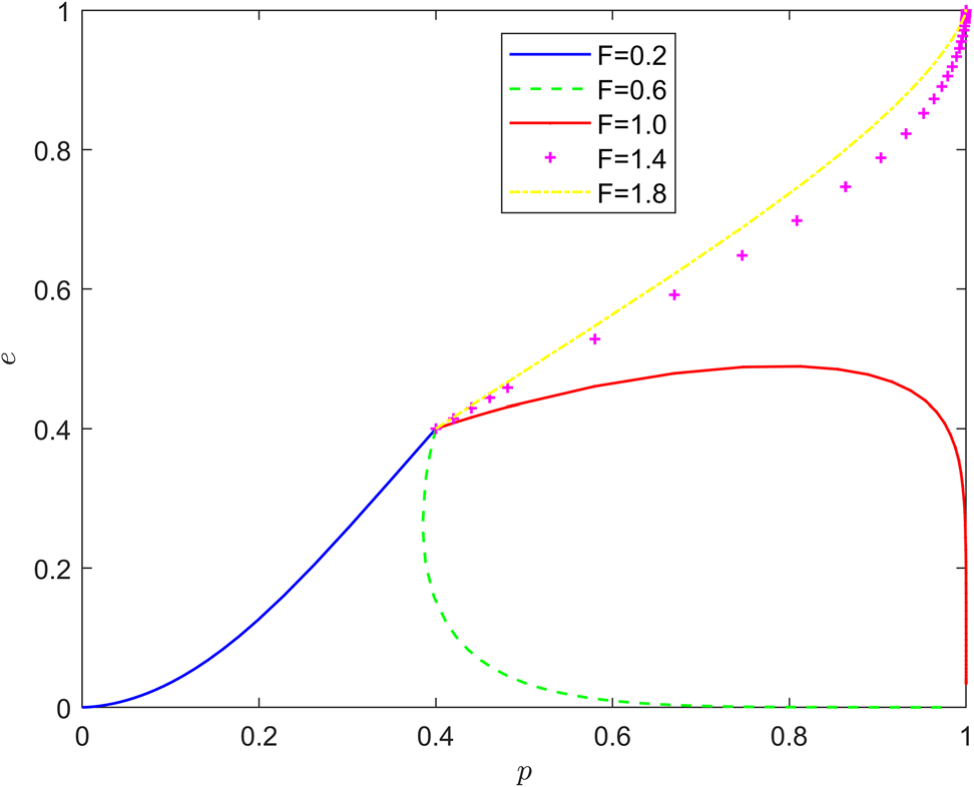
Effect of $F$ on the evolution of the system.

Taking $\left\{ {F = 0.2,F = 0.6,F = 1.0,F = 1.4,F = 1.8} \right\}$, the strategy evolution process and results are shown in Fig. 4.

As can be seen from Figs. 3 and 4, the peer mechanism can significantly affect the battery recycling effect of new energy vehicles, and the peer mechanism can effectively change the position of the system evolution equilibrium point. By observing the horizontal and vertical coordinates of the equilibrium point under different peer mechanisms, it is found that with the increase of incentive intensity and punishment intensity, the equilibrium point of system evolution gradually changes from (0,0) to (1,0), and finally stabilizes around (1,1). Therefore, the introduction of peer mechanism can significantly improve the probability that new energy vehicle manufacturers actively recycle batteries and new energy vehicle retailers actively participate in battery recycling, and promote the evolution of the stability strategy of new energy vehicle battery recycling system to the "Pareto optimal" direction.

#### Impact of feedback mechanism

**Figure 5:**
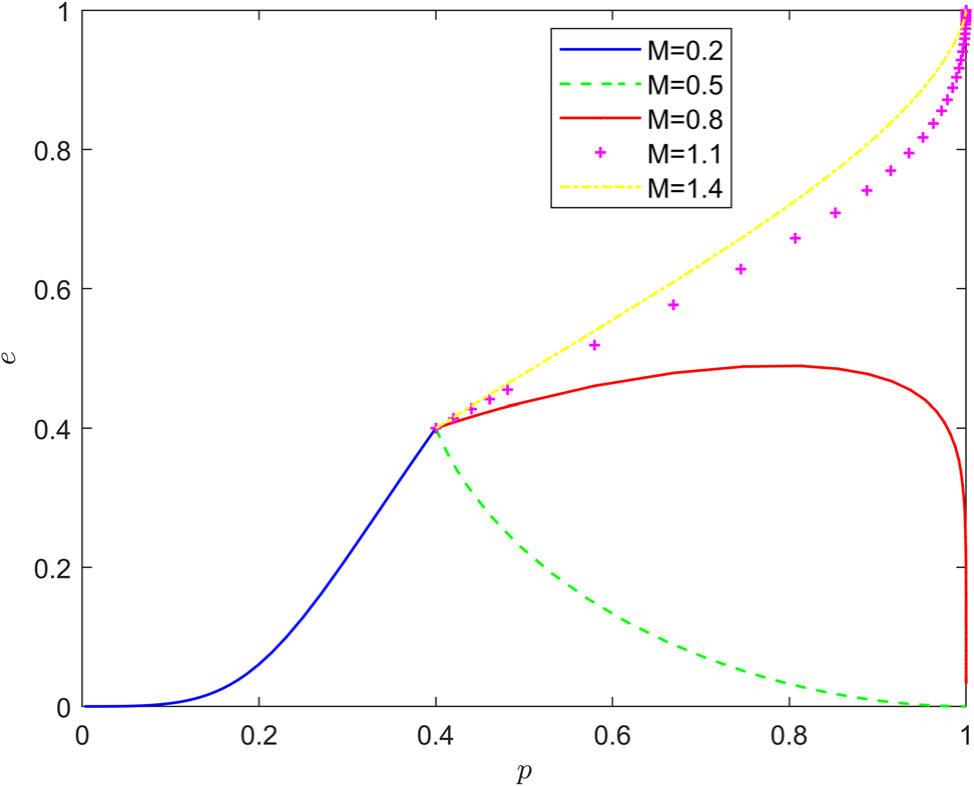
Effect of $M$ on the evolution of the system.

Taking $\left\{ {M = 0.2,M = 0.5,M = 0.8,M = 1.1,M = 1.4} \right\}$, the strategy evolution process and results are shown in Fig. 5.

**Figure 6:**
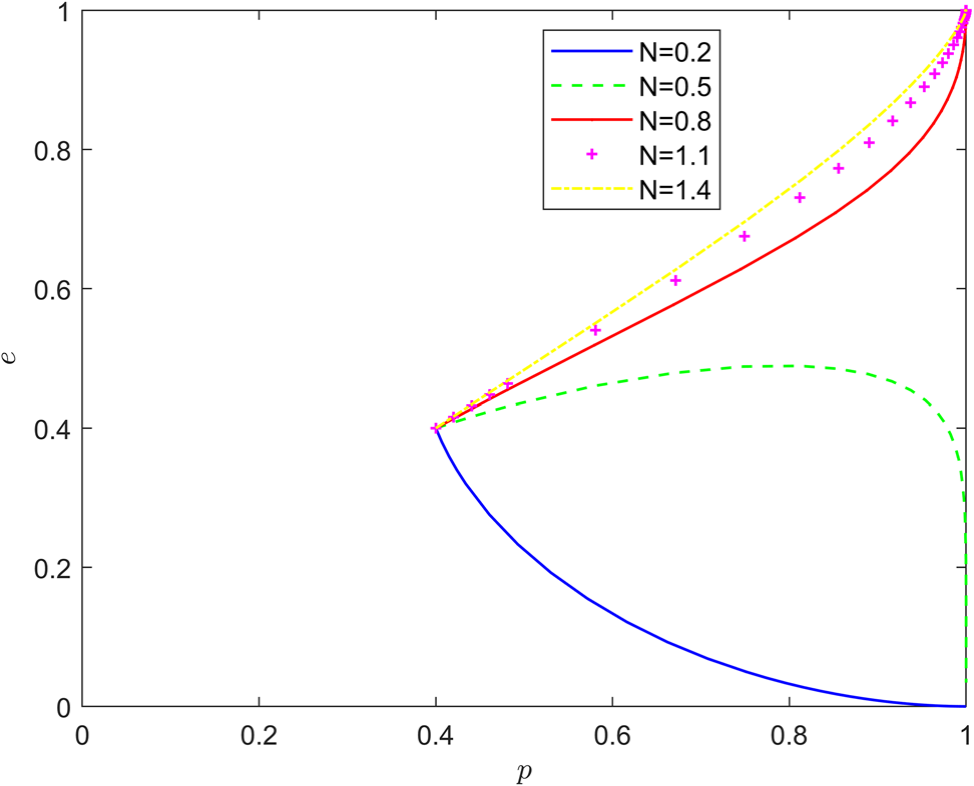
Effect of $N$ on the evolution of the system.

Taking $\left\{ {N = 0.2,N = 0.5,N = 0.8,N = 1.1,N = 1.4} \right\}$, the strategy evolution process and results are shown in Fig. 6.

As can be seen from Figs. 5 and 6, the feedback mechanism can significantly affect the battery recycling effect of new energy vehicles, and the effect of positive feedback mechanism is better than that of negative feedback mechanism. By observing the horizontal and vertical coordinates of the equilibrium point under different feedback mechanisms, it is found that under the positive feedback mechanism, with the strengthening of the feedback mechanism, the equilibrium point of the system evolution gradually changes from (0,0) to (1,0), and finally stabilizes around (1,1). Under the negative feedback mechanism, with the strengthening of the feedback mechanism, the equilibrium point of system evolution gradually changed from (1,0) to (1,1). Therefore, the introduction of feedback mechanism can significantly improve the probability that new energy vehicle manufacturers actively recycle batteries and new energy vehicle retailers actively participate in battery recycling, and promote the evolution of the stability strategy of new energy vehicle battery recycling system to the "Pareto optimal" direction.4.1.4 Impact of risk mechanisms.

**Figure 7:**
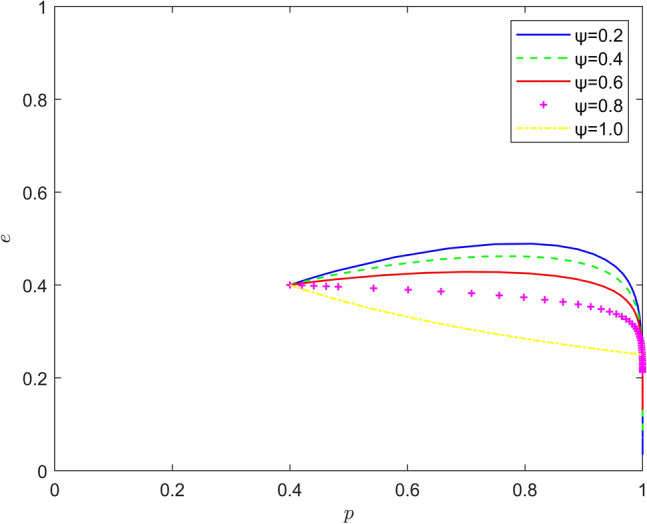
Effect of $\psi$ on the evolution of the system.

Taking $\left\{ {\psi = 0.2,\psi = 0.4,\psi = 0.6,\psi = 0.8,\psi = 1.0} \right\}$, the strategy evolution process and results are shown in Fig. 7.

**Figure 8:**
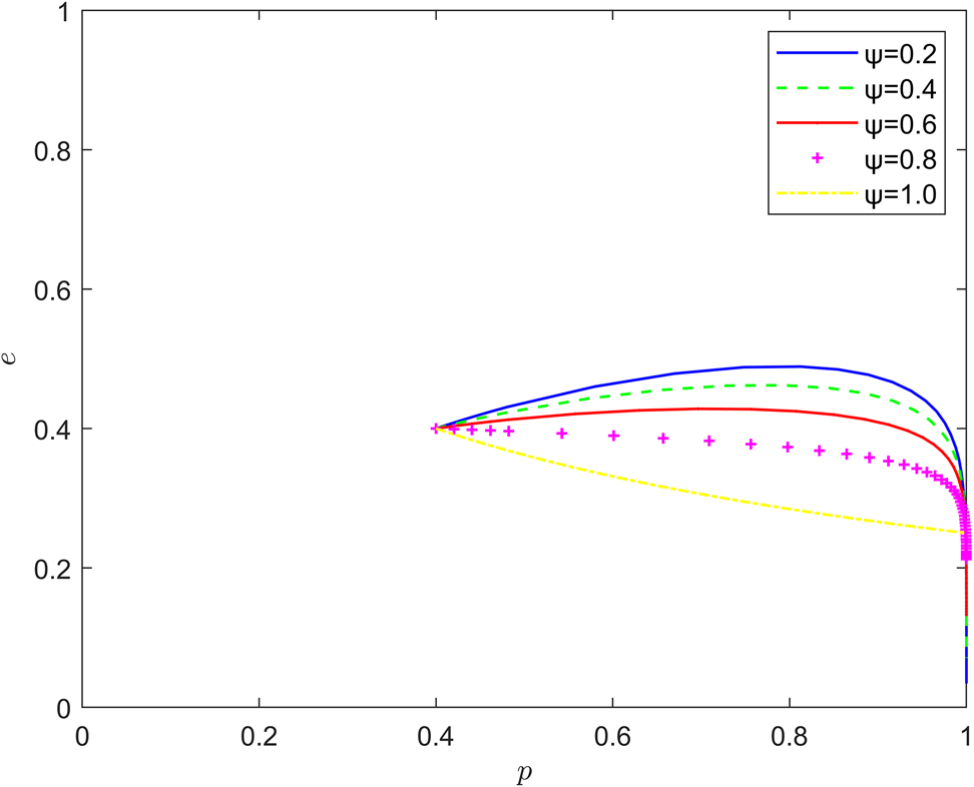
Effect of $\varphi$ on the evolution of the system.

Taking $\left\{ {\varphi = 0.2,\varphi = 0.4,\varphi = 0.6,\varphi = 0.8,\varphi = 1.0} \right\}$, the strategy evolution process and results are shown in Fig. 8.

**Figure 9:**
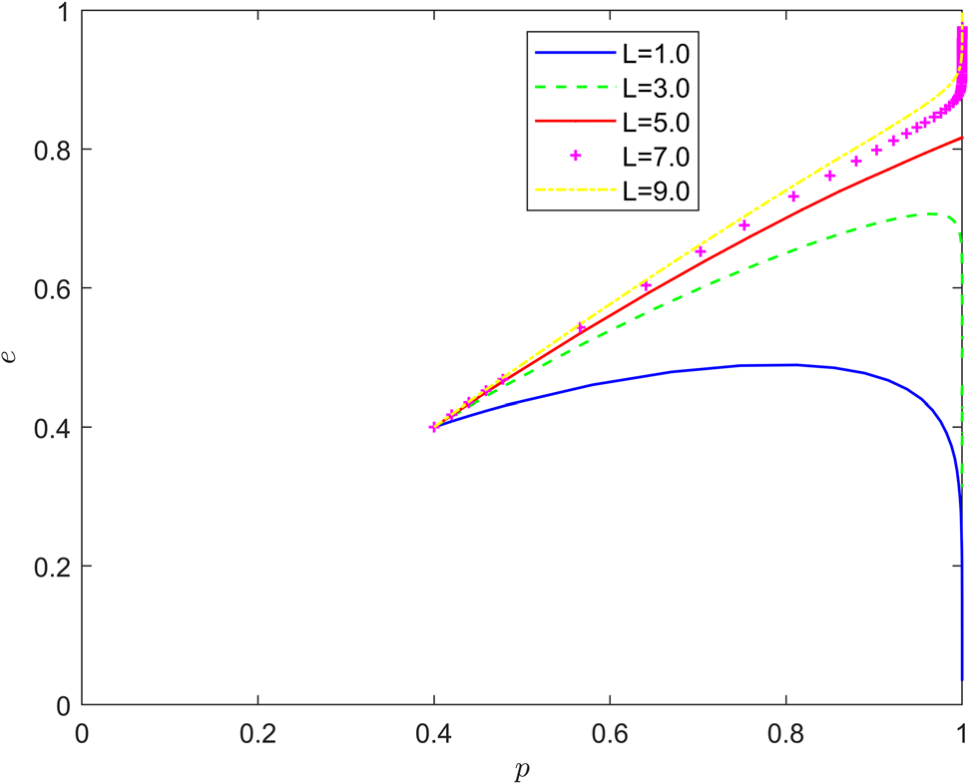
Effect of $L$ on the evolution of the system.

Taking $\left\{ {L = 1.0,L = 3.0,L = 5.0,L = 7.0,L = 9.0} \right\}$, the strategy evolution process and results are shown in Fig. 9.

From Figs. 7, 8 and 9, it can be seen that the risk mechanism can significantly influence the effect of new energy vehicle battery recycling, and the effectiveness of the role of different key elements varies. In terms of risk coefficient, as the risk coefficient increases, the equilibrium point of system evolution shifts to a certain extent in the vertical direction, and the probability of active participation of new energy vehicle retailers in battery recycling is increased, and the increase in the willingness of new energy vehicle retailers to participate in battery recycling is mainly due to the increase in the probability of quality risk. In terms of risk transfer coefficient, as the risk transfer coefficient increases, the probability of new energy vehicle retailers actively participating in battery recycling also increases, and the increase is greater than the increase in the probability under the influence of risk coefficient, and the increase in the willingness of new energy vehicle retailers to participate in battery recycling is mainly due to the transfer of risk. In terms of risk cost, the equilibrium point of system evolution gradually changes from (1,0) to (1,1) with the increase of risk cost, and the increase of new energy vehicle retailers' willingness to participate in battery recycling mainly comes from the increase of potential risk cost.

### Influence of heterogeneous emotions

#### (Rational, rational) state analysis

**Figure 10:**
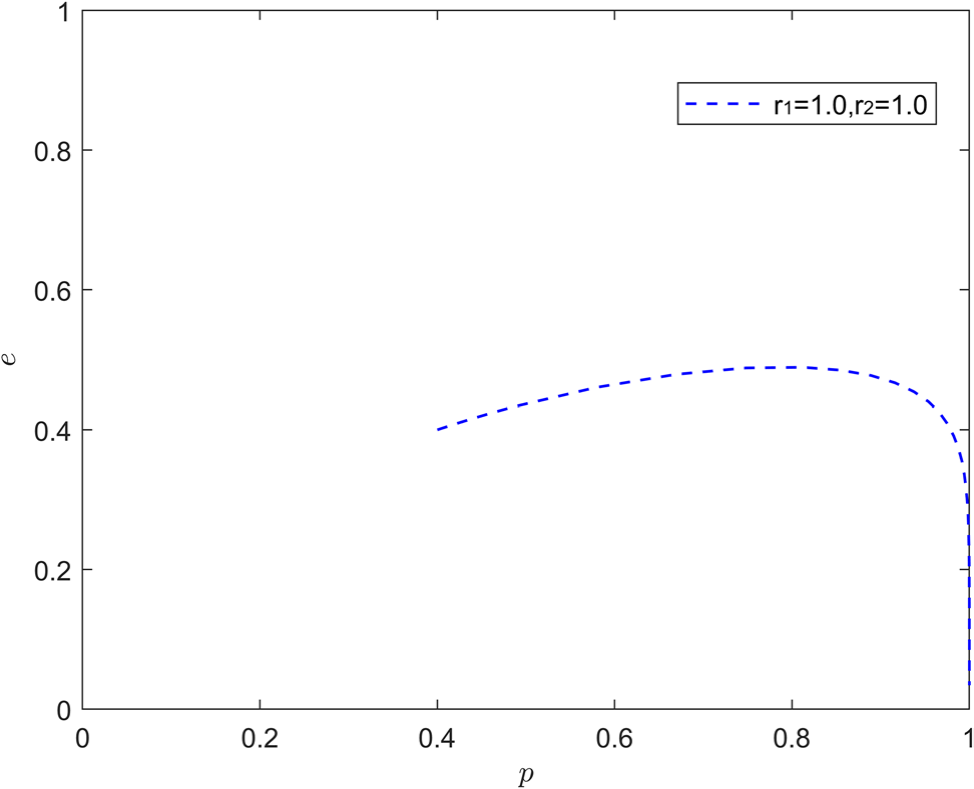
Evolution of the game strategy in the (rational, rational) state.

Figure 10 reflects the equilibrium strategy when the new energy vehicle manufacturer is rational and the new energy vehicle retailer is rational. When $r_{1}{ = }1,r_{2}{ = }1$, the system evolutionary stability point is (1,0), i.e., the new energy vehicle manufacturer chooses active battery recycling and the new energy vehicle retailer chooses negative synergy. At this point, the increase of battery recycling of new energy vehicles mainly comes from the investment of new energy vehicle manufacturers in battery recycling, and the new energy vehicle retailers can get most of the incremental benefits due to the spillover effect, and the new energy vehicle retailers in the downstream of new energy vehicles will enjoy the positive externality at no cost by "riding on the bandwagon". The downstream retailers of new energy vehicles will enjoy positive externalities at no cost.

#### (Optimistic, optimistic) state analysis

**Figure 11:**
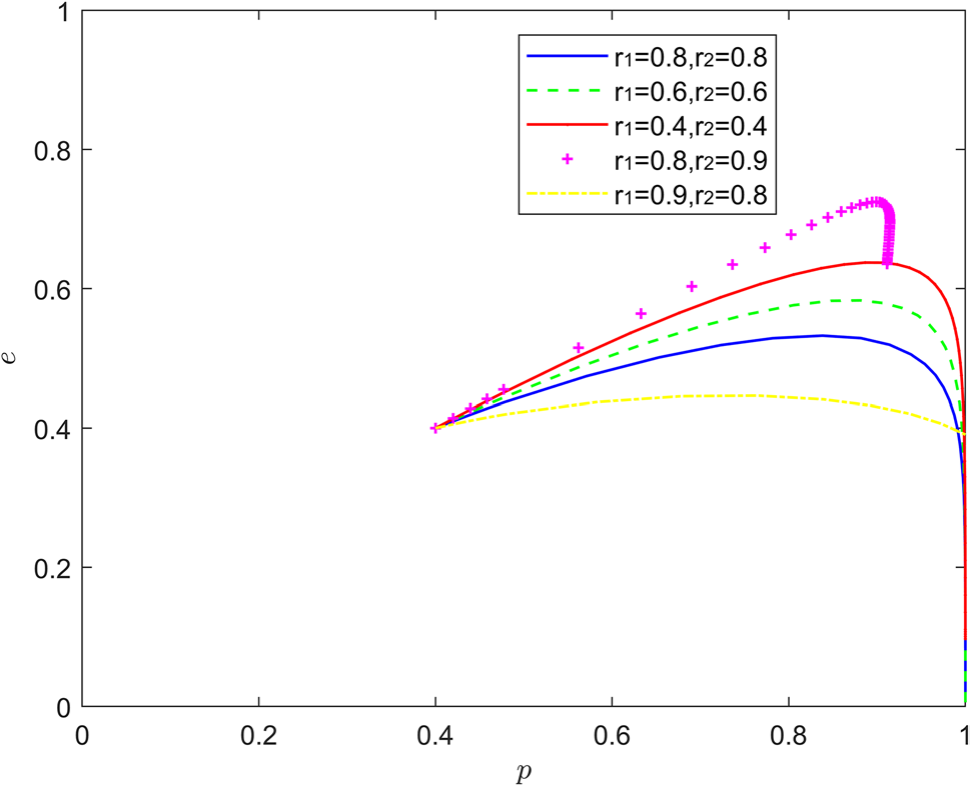
Evolution of the game strategy in the (optimistic, optimistic) state.

Figure 11 reflects the equilibrium strategy when new energy vehicle manufacturers are optimistic and new energy vehicle retailers are optimistic, that is, when $r_{1} < 1,r_{2} < 1$. When the optimism of new energy automobile manufacturers and new energy automobile retailers deepens year-on-year ($r_{1}{ = }r_{2}$), the stable state of system evolution does not change, and the convergence rate of the system gradually slows down. When there is a difference in the optimism intensity between them, a new Nash equilibrium point of mixed strategy appears in the system evolution. When the optimism of new energy automobile manufacturers is stronger than that of new energy automobile retailers ($r_{1} < r_{2}$), the equilibrium point of system evolution moves from the lower right corner of the coordinate area to the upper right corner of the coordinate area. When the optimism of new energy automobile retailers is stronger than that of new energy automobile manufacturers ($r_{1} > r_{2}$), the equilibrium point of system evolution moves upward from the lower right corner of the coordinate area, and the probability of new energy automobile retailers actively cooperating increases to about 0.4. When the optimism of new energy automobile manufacturers is deeper, new energy automobile manufacturers will release more positive signals, and the willingness of new energy automobile retailers to participate in battery recycling will also increase. The above conclusions show that due to the heterogeneity of enterprises, the combination of optimism with different intensity is better than the combination of optimism with the same intensity in promoting the battery recovery of new energy vehicles.

#### (Pessimistic, pessimistic) state analysis

**Figure 12:**
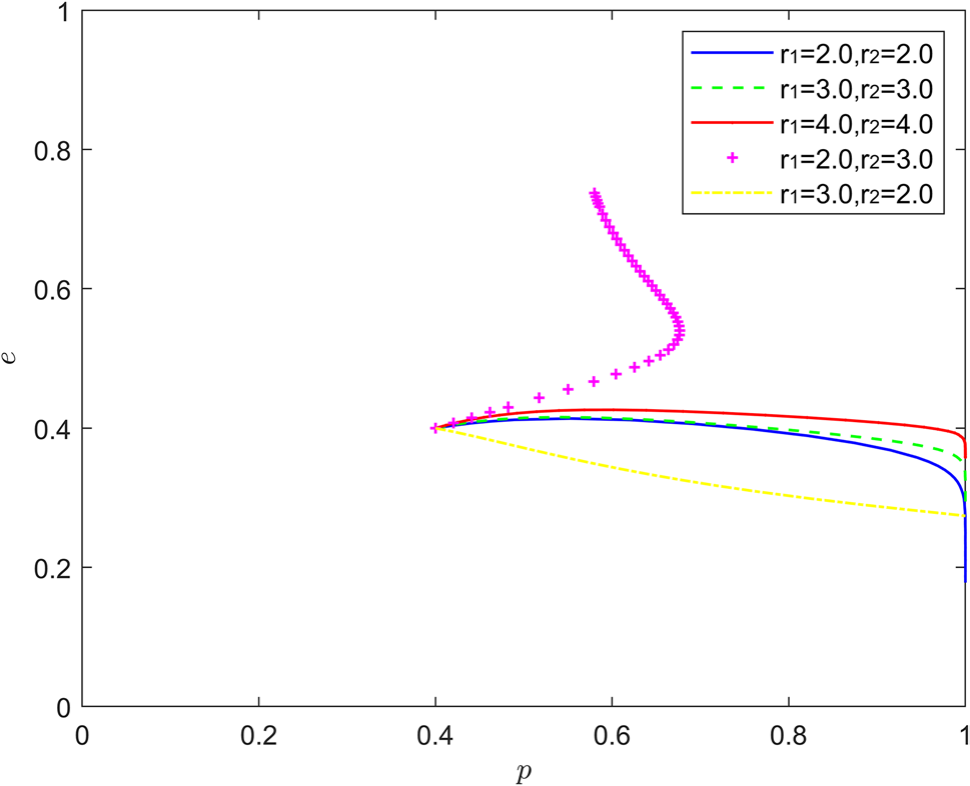
Evolution of the game strategy in the (pessimistic, pessimistic) state.

Figure 12 reflects the equilibrium strategy when the new energy vehicle manufacturer is pessimistic and the new energy vehicle retailer is pessimistic, i.e., when $r_{1} > 1,r_{2} > 1$. When the pessimism of new energy vehicle manufacturers and new energy vehicle retailers deepens year-on-year ($r_{1}{ = }r_{2}$), the steady state of the system evolution does not change and the convergence rate of the system gradually becomes slower. When there is a difference in the intensity of pessimism between the two, a new mixed strategy Nash equilibrium point emerges in the system evolution. When the pessimism of the new energy vehicle manufacturer is stronger than that of the new energy vehicle retailer ($r_{1} > r_{2}$), the equilibrium point of the system evolution shifts upward from the lower right corner of the coordinate region, and the probability of positive synergy of the new energy vehicle retailer increases to about 0.3. When the pessimism of new energy vehicle retailers is stronger than the pessimism of new energy vehicle manufacturers ($r_{1} < r_{2}$). The equilibrium point of the system evolution moves from the lower right corner of the coordinate region to the upper right corner of the coordinate region. When the pessimism of the new energy vehicle retailer is deeper, the more the new energy vehicle retailer does not trust the effectiveness of the new energy vehicle manufacturer's battery recycling, and the new energy vehicle retailer will choose more negative synergy out of the pursuit of their own interests. The above findings suggest that due to the existence of firm heterogeneity, the combination of different intensity of pessimism is better than the combination of the same intensity of pessimism in promoting new energy vehicle battery recycling.

#### (Optimistic, pessimistic) state analysis

**Figure 13:**
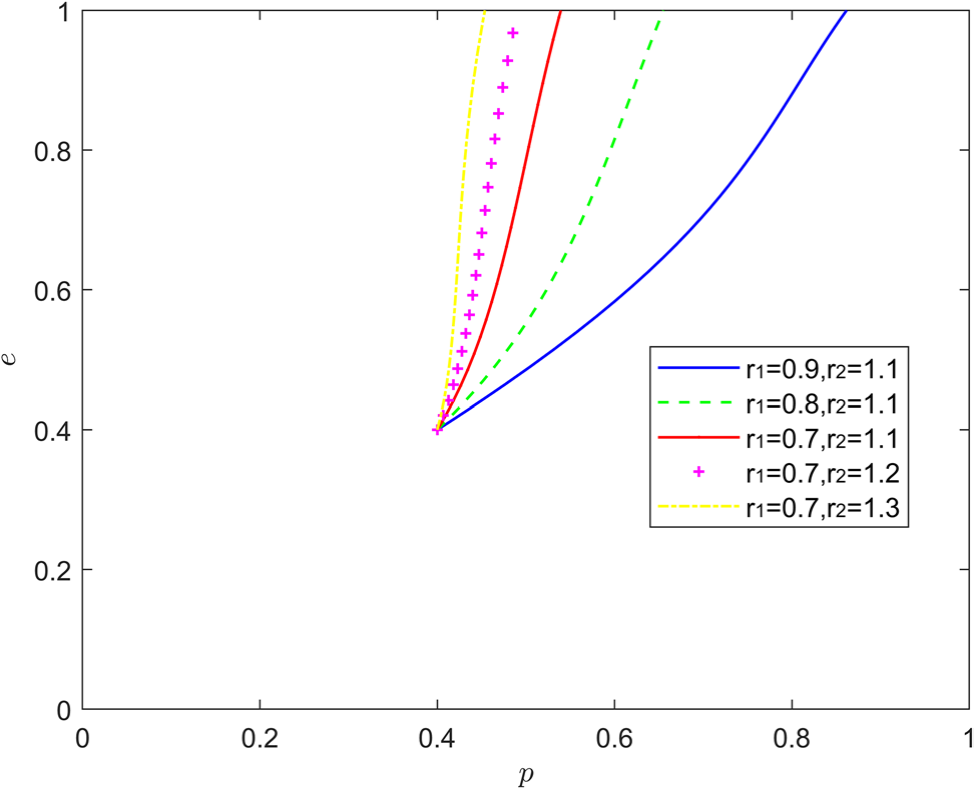
Evolution of the game strategy in the (optimistic, pessimistic) state.

Figure 13 reflects the equilibrium strategy when the new energy vehicle manufacturer is optimistic and the new energy vehicle retailer is pessimistic, i.e., when $r_{1} < 1,r_{2} > 1$. When the new energy vehicle manufacturer remains moderately optimistic and the new energy vehicle retailer remains moderately pessimistic, the equilibrium point of the system evolution moves from the lower right corner of the coordinate region to the upper left corner of the coordinate region, at this time the new energy vehicle manufacturer, which is optimistic about battery recycling, is actively engaged in battery recycling while also releasing positive signals, while the new energy vehicle retailer, which is in pessimistic mood, does not fully believe in the signals transmitted by the new energy vehicle manufacturer, and the new energy vehicle retailer will more actively participate in battery recycling to further protect its own interests. The pessimistic new energy vehicle retailers are not fully convinced by the signals sent by the new energy vehicle manufacturers, and the new energy vehicle retailers will participate more actively in battery recycling to further protect their own interests. When the optimism of new energy vehicle manufacturers or the pessimism of new energy vehicle retailers is too deep, the equilibrium point of system evolution will move horizontally towards the upper left corner of the coordinate region, and the probability of new energy vehicle retailers actively participating in battery recycling will be significantly reduced. Therefore, moderate optimism of new energy vehicle manufacturers and moderate pessimism of new energy vehicle retailers will help new energy vehicle battery recycling, while too high optimism and pessimism will inhibit this driving effect.

#### (pessimistic, optimistic) state analysis

**Figure 14:**
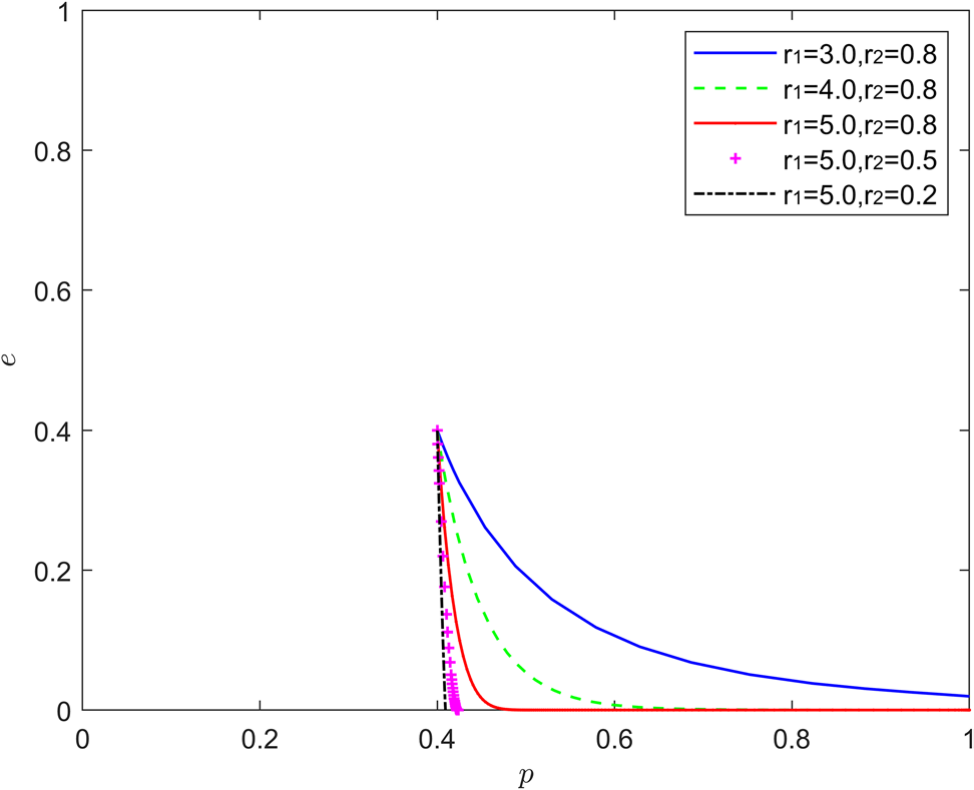
Evolution of the game strategy in the (pessimistic, optimistic) state.

Figure 14 reflects the equilibrium strategy when the new energy vehicle manufacturer is pessimistic and the new energy vehicle retailer is optimistic, i.e., when $r_{1} > 1,r_{2} < 1$. (The heterogeneous combination of (pessimism, optimism) does not change the overall trend of system evolution. As the pessimism of the new energy vehicle manufacturers and the optimism of the new energy vehicle retailers deepen, the probability of the new energy vehicle manufacturers choosing positive battery recycling decreases significantly, and the strategy choice of the new energy vehicle retailers stabilizes in negative synergy, and the rate of stabilization of the strategy choice keeps increasing. (This heterogeneous combination of emotions (pessimism, optimism) has a significant inhibitory effect on new energy vehicle battery recycling, with pessimism of new energy vehicle manufacturers reducing their willingness to recycle batteries, and optimism of new energy vehicle retailers causing them to overly trust new energy vehicle manufacturers to recycle batteries and thus choose inaction more often.

#### (Emotional, rational) state analysis

**Figure 15:**
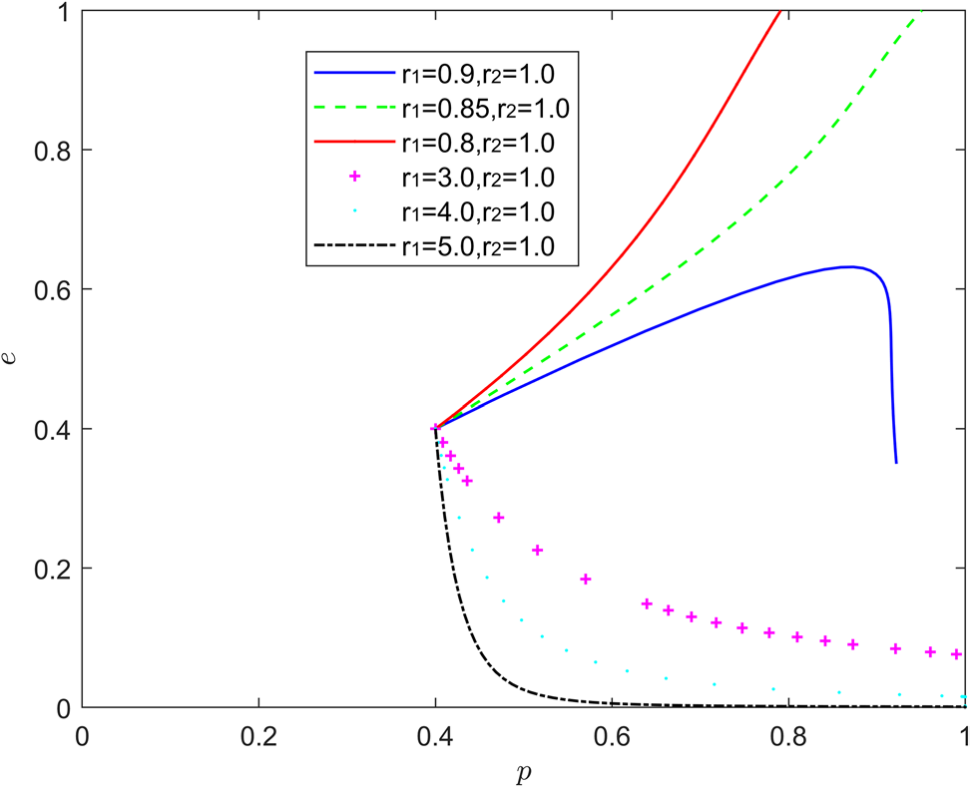
Evolution of the game strategy in the (emotional, rational) state.

Figure 15 reflects the equilibrium strategy when the new energy vehicle manufacturer is emotional and the new energy vehicle retailer is rational, i.e., when $r_{1} \ne 1,r_{2} = 1$. When the new energy vehicle manufacturer is optimistic and the new energy vehicle retailer is rational ($r_{1} > 1,r_{2} = 1$), the equilibrium point of system evolution gradually moves from the lower right corner of the coordinate region to the upper left corner of the coordinate region, but it is worth noting that the relationship between the strength of the new energy vehicle manufacturer's optimism and positive battery recovery is nonlinear. When new energy vehicle manufacturers are pessimistic and new energy vehicle retailers are rational ($r_{1} < 1,r_{2} = 1$), the rate at which the system evolution converges to the equilibrium of (1,0) increases significantly as the pessimism of new energy vehicle manufacturers deepens. It can be seen that the moderate optimism of new energy vehicle manufacturers and the rationality of new energy vehicle retailers help new energy vehicle battery recycling, when new energy vehicle manufacturers maintain a high level of investment in battery recycling and a positive attitude, while rational new energy vehicle retailers will continuously strengthen their support and assistance to new energy vehicle manufacturers in battery recycling, so as to better enjoy the positive Spillover effect, but the intensity of optimism is not the greater the better or the smaller the better, but to maintain a moderate range of intervals. Therefore, it is important to be alert to the inhibiting effects of pessimism and "non-moderate range" optimism on battery recycling.

#### (Rational, emotional) state analysis

**Figure 16:**
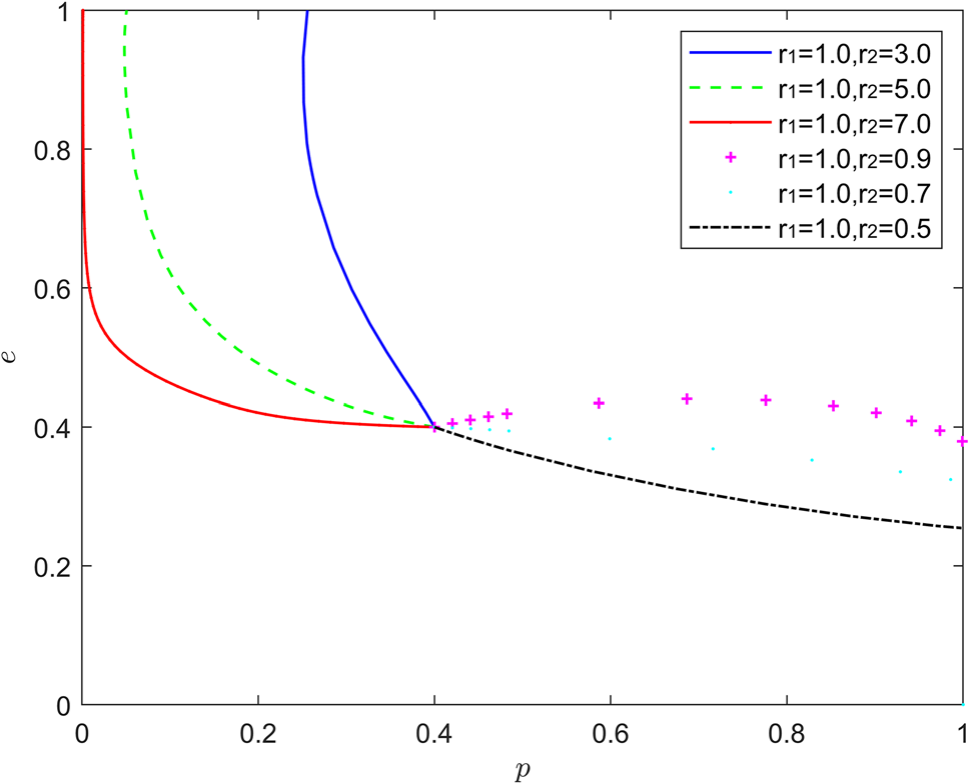
Evolution of the game strategy in the (rational, emotional) state.

Figure 16 reflects the equilibrium strategy when the new energy vehicle manufacturer is rational and the new energy vehicle retailer is optimistic, i.e., when $r_{1}{ = }1,r_{2} \ne 1$. When the new energy vehicle manufacturers are rational and the new energy vehicle retailers are pessimistic ($r_{1} = 1,r_{2} > 1$), the equilibrium point of the system evolution gradually changes from (1,0) to (0,1). When the new energy vehicle manufacturer is rational and the new energy vehicle retailer is optimistic ($r_{1} = 1,r_{2} < 1$), the equilibrium point of the system evolution moves upward parallel to the coordinate axis, and the strategy choice of the new energy vehicle manufacturer remains unchanged, and the probability of the new energy vehicle retailer to participate in battery recycling increases slightly. Thus, the pessimism of the new energy vehicle retailers increases their probability of participating in battery recycling, while the rational new energy vehicle manufacturers can choose to "piggyback" to save their own costs. Optimistic new energy vehicle retailers are also more likely to participate in battery recycling by amplifying the signals sent by new energy vehicle manufacturers.

## Conclusion

This paper combines RDEU theory with evolutionary game theory to investigate how to achieve optimal steady state in new energy vehicle battery recycling when considering heterogeneous emotions of decision makers. The study shows that:

## Research conclusions

1. In the new energy vehicle battery recycling system, the battery recycling is often in a non-coordinated state due to the fact that there is no unanimous cooperation between multiple actors, which leads to a non-Pareto-optimal evolution trend in the system evolution. There are two types of key factors affecting the recycling of new energy vehicle batteries. One is external factors, such as government policies, industry regulations, market environment, etc., which together constitute the external framework of new energy vehicle battery recycling. The other category is internal factors, which mainly refer to the cost and benefit trade-offs of battery recycling between new energy vehicle manufacturers and new energy vehicle retailers. The regulation of these two types of factors is the key to influencing the battery recycling strategies of new energy vehicle manufacturers and the synergistic strategies of new energy vehicle retailers.
2. The effect of altruistic preference on new energy vehicle battery recycling is nonlinear, which makes the altruistic preference of new energy vehicle manufacturers and new energy vehicle retailers better exert its positive effect on new energy vehicle battery recycling only when they are in the moderate range. The peer mechanism can effectively change the location of the equilibrium point of the system evolution, which in turn can significantly affect the effect of new energy vehicle battery recycling. Feedback mechanism can significantly affect the effect of new energy vehicle battery recycling, and the effectiveness of positive feedback mechanism is better than that of negative feedback mechanism. In addition, as for the risk mechanism, the risk factor, risk transfer coefficient and risk cost can significantly improve the effect of new energy vehicle battery recycling, and the effectiveness of risk cost is better than that of risk factor and risk transfer coefficient.
3. Emotions, an irrational factor, can significantly change the stability of the evolution of the new energy vehicle battery recycling system by influencing the behavioral decisions of decision makers, and heterogeneous emotions have different effects on the evolution of the system. (The combination of (optimism of new energy vehicle manufacturers, pessimism of new energy vehicle retailers) and (optimism of new energy vehicle manufacturers, rationality of new energy vehicle retailers) is more effective than other emotion combinations, and the motivation of new energy vehicle manufacturers to recycle batteries and the degree of positive signals they send are higher in the above context. The driving force is also higher, and it is easier to achieve an optimal and stable state for new energy vehicle battery recycling.

### Managerial implications

Based on the above research conclusions, the following Managerial implications can be obtained:

1. New energy vehicle battery recycling can realize the optimal recycling steady state by establishing external norms and regulating subjective preferences. On the one hand, it is necessary to improve relevant laws and regulations to provide a legal basis for new energy vehicle battery recycling, and to create a healthy development environment and a low-carbon atmosphere for new energy vehicles through good external norms. In addition, government regulators should also strengthen the matching of basic policy tools with the recycling industry chain, and implement the policy instrument combination of “one place, one policy” based on different real-life situations^39^. On the other hand, new energy vehicle manufacturers and demanders should establish an economic partnership to form a community of interest and social responsibility, to ensure that waste battery recycling enterprises make profits, and to enhance the intrinsic driving force of new energy vehicle battery recycling.
2. Government regulators should emphasize the role of incentive policies and give policy support to relevant subjects. Government regulators and relevant industry associations should establish a comprehensive peer mechanism, giving full play to the promotion of peer incentives and the disciplinary role of peer penalties. Government regulators should also strengthen the consumer feedback mechanism to support battery recycling. Strengthen the correct perception of risk, enhance risk prevention awareness and enhance altruistic preference of the main actors involved in the measures can effectively promote the new energy vehicle battery recycling to achieve a steady state. However, it is also necessary to be vigilant against the inhibiting effect of "free ride" and "non-moderate zone" altruistic preference on battery recycling. In addition, the government should strengthen cooperation with the market, utilize blockchain technology and other emerging technologies to continuously innovate incentive mechanisms and crack down on unqualified recyclers^40^.
3. For new energy vehicle manufacturers, an optimistic atmosphere should be created, and favorable information on battery recycling should be actively released to enhance the self-confidence of new energy vehicle manufacturers in the long-term returns of battery recycling. For new energy vehicle retailers, a rational or pessimistic atmosphere should be created, and negative signals should be released through various channels to strengthen the awareness of new energy vehicle retailers of the severe situation of battery recycling in the context of the "scrapping wave". In addition, the government should strengthen cooperation with academia, establish an industry-academia-research platform, conduct in-depth research on the impact of decision-makers' emotions on battery recycling, and strengthen the role of emotional mechanisms in supporting and guiding industrial practices.

The model designed in this paper mainly discusses the behavioral evolution between new energy vehicle manufacturers and new energy vehicle retailers, but there are multiple recycling modes of new energy vehicle battery recycling. Therefore, it would be a valuable research direction to construct an evolutionary game model based on other recycling modes with the participation of multiple subjects, and to study the influence of heterogeneous emotions on the evolutionary behavior of multiple subjects.

## Data Availability

Data can be obtained from the corresponding author upon reasonable request.

## References

[1] Zhang Y, Lu M, Shen S (2020). On the values of vehicle-to-grid electricity selling in electric vehicle sharing. Manuf. Serv. Oper. Manag..

[2] Liu Q, Wen X, Peng H, Cao Q (2023). Key technology breakthrough in new energy vehicles: Configuration path evolution from innovative ecosystem perspective. J. Clean. Prod..

[3] Peng H, Xiao Z, Wang M, Wang X, Wang J (2023). An integrated decision support framework for new energy vehicle evaluation based on regret theory and QUALIFLEX under Z-number environment. Inf. Sci..

[4] Ma Y (2018). Comprehensive policy evaluation of NEV development in China, Japan, the United States, and Germany based on the AHP-EW model. J. Clean. Prod..

[5] Mousavi M, Gitinavard H, Mousavi SM (2017). A soft computing based-modified ELECTRE model for renewable energy policy selection with unknown information. Renew. Sustain. Energy Rev..

[6] Tang Y, Zhang Q, Li Y, Wang G, Li Y (2018). Recycling mechanisms and policy suggestions for spent electric vehicles' power battery—A case of Beijing. J. Clean. Prod..

[7] Zheng C (2023). Power battery third-party reverse logistics provider selection: Fuzzy evidential reasoning. Energy Environ..

[8] Lin Y, Yu Z, Wang Y, Goh M (2023). Performance evaluation of regulatory schemes for retired electric vehicle battery recycling within dual-recycle channels. J. Environ. Manag..

[9] Wu Y, Yang L, Tian X, Li Y, Zuo T (2020). Temporal and spatial analysis for end-of-life power batteries from electric vehicles in China. Resour. Conserv. Recycl..

[10] Huang J, Wen J, He F (2023). Research on evaluation of power battery recycling efficiency of new energy vehicle based on DEA. Ind. Eng. Innov. Manag..

[11] Asit T, Atanu B, Padhy RK, Sachin KM, Roopendra R (2023). Drivers of lithium-ion batteries recycling industry toward circular economy in industry 4.0. Comput. Ind. Eng..

[12] Zhao S (2021). Unveiling the recycling characteristics and trends of spent lithium-ion battery: a scientometric study. Environ. Sci. Pollut. Res..

[13] dos Santos MP (2021). A technology for recycling lithium-ion batteries promoting the circular economy: The RecycLib. Resour. Conserv. Recycl..

[14] Yao P (2021). The role of nickel recycling from nickel-bearing batteries on alleviating demand-supply gap in China's industry of new energy vehicles. Resour. Conserv. Recycl..

[15] Zhang Z, Wang X, Su C, Sun L (2022). Evolutionary game analysis of shared manufacturing quality synergy under dynamic reward and punishment mechanism. Appl. Sci..

[16] Wei L, Wang C, Li Y (2022). Governance strategies for end-of-life electric vehicle battery recycling in China: A tripartite evolutionary game analysis. Front. Environ. Sci..

[17] Zhang H (2022). Waste battery-to-reutilization decisions under government subsidies: An evolutionary game approach. Energy.

[18] He L, Sun B (2021). Exploring the EPR system for power battery recycling from a supply-side perspective: An evolutionary game analysis. Waste Manag..

[19] Guo S, Liu G, Guo X, Wang Y (2022). Game evolution and simulation analysis of power battery recycling in China under conflicting supply and demand of critical metals. Front. Energy Res..

[20] Chateauneuf A, Cohen M, Meilijson I (2005). More pessimism than greediness: A characterization of monotone risk aversion in the rank-dependent expected utility model. Econ. Theory.

[21] Lerner JS, Li Y, Valdesolo P, Kassam KS (2015). Emotion and decision making. Ann. Rev. Psychol..

[22] Sabine R (2012). Risk communication, public engagement, and climate change: A role for emotions. Risk Anal..

[23] Chu H, Yang JZ (2019). Emotion and the psychological distance of climate change. Sci. Commun..

[24] Dong J (2022). Promoting dynamic pricing implementation considering policy incentives and electricity retailers’ behaviors: An evolutionary game model based on prospect theory. Energy Policy.

[25] Quiggin J (1991). Comparative statics for rank-dependent expected utility theory. J. Risk Uncertain..

[26] Liu J, Lyu Y, Zhao H, Chen J (2021). Game analysis of nuclear wastewater discharge under different attitudes: Seeking a potential equilibrium solution. Sci. Total Environ..

[27] Xinping W (2022). Game analysis of the evolution of energy structure transition considering low-carbon sentiment of the decision-makers in the context of carbon neutrality. Processes.

[28] Zhang Z, Wang X, Su C, Sun L (2022). Evolutionary game analysis of shared manufacturing quality innovation synergetic behavior considering a subject’s heterogeneous emotions. Processes.

[29] Wenlong L, Shupei H, Yabin Q, Haizhong A (2022). RDEU hawk-dove game analysis of the China-Australia iron ore trade conflict. Resour. Policy.

[30] Wang X, Zhang Z, Guo Z, Su C, Sun L (2023). Energy structure transformation in the context of carbon neutralization: Evolutionary game analysis based on inclusive development of coal and clean energy. J. Clean. Prod..

[31] Smith JM, Price GR (1973). Logic of animal conflict. Nature.

[32] Hofbauer J, Schuster P, Sigmund K (1979). Evolutionary stable strategies and game dynamics. J. Theor. Biol..

[33] Kang K, Bai L, Zhang J (2023). A tripartite stochastic evolutionary game model of complex technological products in a transnational supply chain. Comput. Ind. Eng..

[34] Deng J (2024). Evolutionary game analysis of chemical enterprises' emergency management investment decision under dynamic reward and punishment mechanism. J. Loss Prev. Process Ind..

[35] Li F, Guo Y, Dong T, Liu B, Geng X (2024). Tripartite evolutionary game analysis on corporate carbon reduction decisions considering dual supervision under carbon trading. Comput. Ind. Eng..

[36] Yue X, Khan DS, Zhao S, Li F (2023). An evolutionary game for the behavior of third-party evaluators in pension public–private partnership incorporating public participation. Sci. Rep..

[37] Cao X, Li C (2020). evolutionary game simulation of knowledge transfer in industry-university-research cooperative innovation network under different network scales. Sci. Rep..

[38] John Q (1982). A theory of anticipated utility. Journal of Economic Behavior & Organization.

[39] Zhang H (2020). Echelon utilization of waste power batteries in new energy vehicles: Review of Chinese policies. Energy.

[40] Li Z, Zhong X, Xu X (2023). The mechanism of retired power batteries recycling through blockchain token incentives. J. Clean. Prod..

